# Activation of mesenchymal stem cells by macrophages promotes tumor progression through immune suppressive effects

**DOI:** 10.18632/oncotarget.8064

**Published:** 2016-03-14

**Authors:** Xiao-hua Jia, Guo-wei Feng, Zhong-liang Wang, Yang Du, Chen Shen, Hui Hui, Dong Peng, Zong-jin Li, De-ling Kong, Jie Tian

**Affiliations:** ^1^ Key Laboratory of Molecular Imaging of the Chinese Academy of Sciences, Institute of Automation, Chinese Academy of Sciences, Beijing 100190, China; ^2^ Department of Genitourinary Oncology, Tianjin Medical University Cancer Institute and Hospital, National Clinical Research Center for Cancer, Key Laboratory of Cancer Prevention and Therapy, Tianjin 300060, China; ^3^ School of Life Science and Technology, Xidian University, Shaanxi, Xi'an 710071, China; ^4^ Department of Pathophysiology, Nankai University School of Medicine, Tianjin 300071, China; ^5^ State Key Laboratory of Medicinal Chemical Biology, Key Laboratory of Bioactive Materials, Ministry of Education, College of Life Sciences, Nankai University, Tianjin 300071, China; ^6^ Beijing Key Laboratory of Molecular Imaging, Beijing 100190, China

**Keywords:** mesenchymal stem cells, macrophages, inflammation, cancer, MCP1

## Abstract

Cancer development and progression is linked to tumor-associated macrophages (TAMs). Distinct TAMs subsets perform either protective or pathogenic effects in cancer. A protective role in carcinogenesis has been described for M1 macrophages, which activate antitumor mechanisms. By comparison, TAMs isolated from solid and metastatic tumors have a suppressive M2-like phenotype, which could support multiple aspects of tumor progression. Currently, it has not been clearly understood how macrophages in tumor-associated stroma could be hijacked to support tumor growth. Mesenchymal stem cells (MSCs) actively interact with components of the innate immune system and display both anti-inflammatory and pro-inflammatory effects. Here, we tested whether MSCs could favor the tumor to escape from immunologic surveillance in the presence of M1 macrophages. We found that MSCs educated by M1 condition medium (cMSCs) possessed a greatly enhanced ability in promoting tumor growth *in vivo*. Examination of cytokines/chemokines showed that the cMSCs acquired a regulatory profile, which expressed high levels of iNOS and MCP1. Consistent with an elevated MCP1 expression in cMSCs, the tumor-promoting effect of the cMSCs depended on MCP1 mediated macrophage recruitment to tumor sites. Furthermore, IL-6 secreted by the cMSCs could polarize infiltrated TAMs into M2-like macrophages. Therefore, when macrophages changed into M1 pro-inflammation type in tumor microenvironment, the MSCs would act as poor sensors and switchers to accelerate tumor growth.

## INTRODUCTION

Cancers develop in complex tumor microenvironments, which include cells such as endothelial cells, immune cells, tumor-associated macrophages (TAMs), and mesenchymal stem cells (MSCs) [1]. Non-cancerous stromal cells have different capabilities to induce both tumor-promoting and tumor-antagonizing effects [2]. The contradictory phenomenon brings us a problem how the tumor-associated stroma at the primary sites is hijacked to support tumor growth. The key mechanisms may focus on the role of macrophages, immune suppressor cells, MSCs, the vasculature and various other components of a tumor-supportive microenvironment.

Macrophages are resident phagocytic cells in tissues, which play important roles in steady-state tissue homoeostasis by removing cellular debris and apoptotic cells. Although macrophages are classically regarded as key effector cells during immune defense, numerous studies have demonstrated a clear role for TAMs in supporting various aspects of tumor development [3]. An explanation for the disparate roles of macrophages during normal tissue homeostasis and carcinogenesis lies in their phenotype [4]. At the extremes of their phenotypic continuum, macrophages range from an M1 to M2 polarization status: classically activated M1 macrophages have an anti-tumorigenic role; on the other hand, alternatively activated M2 macrophages promote anti-inflammatory responses and have pro-tumorigenic functions. Currently, we do not fully understand how macrophages initially switch from being anti-tumor to pro-tumorigenic at the onset of disease [5].

MSCs are one of the major components of the tumor stroma and there is a close interaction between MSCs and the immune system [6–9]. MSCs have been shown to interact with a variety of adaptive immune cells including T cells, B cells and dendritic cells [10]. Recently, it was demonstrated that the MSCs actively interacted with components of the innate immune system and that through these interactions, they displayed both anti-inflammatory and pro-inflammatory effects [11, 12]. Within the innate immune system, it was well established that macrophages were key players in initiating and controlling inflammation and the MSCs could influence macrophage functions [13, 14].

In a tumor microenvironment, the MSCs are often constantly exposed to immune cells and inflammatory cytokines/chemokines. They may have acquired functions that are distinct from those of normal MSCs [15]. The unique features may play a role in regulating the tumor microenvironment and ultimately affecting tumor progression. The aim of this study was to investigate whether the MSCs could favor a tumor to escape from immunologic surveillance in an environment of M1 macrophages. To assess the possible effects of M1 macrophages on the MSCs in tumor growth, we treated MSCs with condition medium derived from M1 macrophages (cMSCs), characterized by their tumor-promoting activity and phenotype, and studied the mechanism of the cMSCs in affecting tumor growth in comparison to normal MSCs. We found that M1 macrophages could activate the MSCs to adopt a regulatory phenotype, and the cMSCs promoted tumor growth by iNOS and MCP1. Therefore, this study revealed that the MSCs sensed and controlled inflammation to promote tumor growth in a pro-inflammatory environment.

## RESULTS

### cMSCs had more potential than untreated-MSCs to promote tumor growth

Macrophage cell-line RAW264.7 cell exposure to IFN-γ and LPS drive M1 polarization with potentiated cytotoxic and antitumoral properties. To confirm the M1 macrophage phenotype, the expression of iNOS (M1 macrophage marker) examined by a FACS analysis clearly increased, while the expression of CD11b (macrophage marker) remained unchanged (Figure S1A). Macrophage M1 polarization was also assessed at the mRNA level using real-time PCR by measuring the expression of several classical M1 markers: TNFα, IL-1β, and iNOS. An increased IL-1β and iNOS expression profile was obtained by incubation with IFN-γ and LPS. We also checked the expression at the mRNA level of several M2 markers (IL-10, Arg-1 and FIZZ-1) in M1 macrophages, but in our conditions, we observed no significant expression of these genes. In addition, the mRNA abundance of signal regulatory protein-a (SIRPα) was significantly reduced (Figure S1A). SIRPα is a membrane receptor expressed by macrophages. The interactions of CD47-SIRPα form a barrier for antibody-mediated tumor cell destruction [16]. In order to study the effects of M1 macrophages on cancer cell response *in vivo*, prior to implantation of M1 macrophages, breast cancer models with MDA-MB-231-FLUC cells were set up. Two hours after M1 macrophages had been injected into tumor sites, we found that M1 macrophages exhibited radical scavenging potential since the amount of tumor cells remaining was 10% compared to the control group (Figure S1B). Therefore, incubation of macrophages with IFN-γ and LPS induces their polarization into M1 macrophages, and M1-polarized macrophages have strong antitumor activity to elicit tumor disruption.

**Figure 1:**
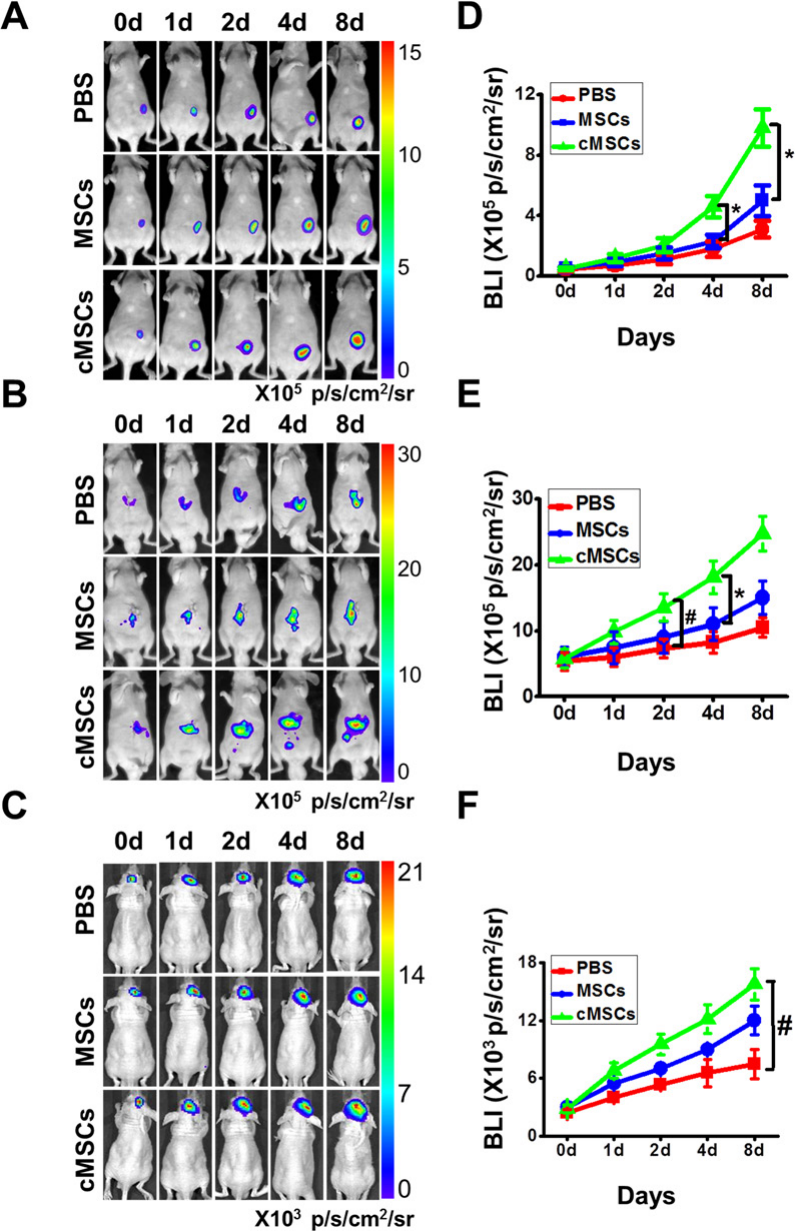
cMSCs promoted tumor growth *in vivo* to a greater extent than untreated-MSCs (**A**–**C**) D-Luciferin induced bioluminescence obtained in breast (**A**), liver (**B**), and brain (**C**) cancer mice co-injected with cMSCs, MSCs or PBS. cMSCs -treated mice displayed a significant increase in tumor growth. (**D**–**F**) Quantification of the bioluminescent signal in corresponding breast (**D**), liver (**E**), and brain (**F**) tumors after treatments. *P < 0.05 vs. MSCs group; ^#^*P* < 0.05 vs. PBS group.

To investigate the characterization of the cMSCs, MSCs were isolated from murine bone marrow. The morphology of the mouse MSCs displayed a homogenous spindle-shaped population. FACS was used to identify the surface marker expression of the MSCs. High expression of CD29, CD73, CD90 and CD105 was observed, but CD34 and CD45 were down-regulated (data not shown). MSC were stimulated by the medium from M1 macrophages for 24 hours to achieve the cMSCs. To determine whether the cMSCs could promote human tumor growth, we injected the cMSCs into immune deficient mice with human carcinoma cells, while untreated MSCs were used as controls. When co-injected with MDA-MB-231-FLUC breast cancer cells, the cMSCs but not the untreated MSC remarkably increased the tumor-initiating ability and tumor growth (Figure 1A, 1D). We also tested the effect of the cMSCs on hepatocellular HCC-LM3-FLUC tumor growth *in vivo*. Similar to the breast cancer model, the cMSCs caused a tumor-promoting effect in the hepatocellular carcinoma model as compared to the untreated control MSCs group (Figure 1B, 1E). The cMSCs also led to a significant tumor promotion of malignant glioblastoma U87MG-FLUC cells (Figure 1C, 1F). Furthermore, after the cMSCs were co-injected with murine 4T1-FLUC cells into a fat pad of normal mice, the cMSCs were more effective than the control MSCs in promoting tumor growth (data not shown). Therefore, the cMSCs displayed greater potential to promote tumor growth in comparison to the untreated MSCs.

### Stimulated by M1 medium altering the cytokine/chemokine expression in the cMSCs

**Figure 2:**
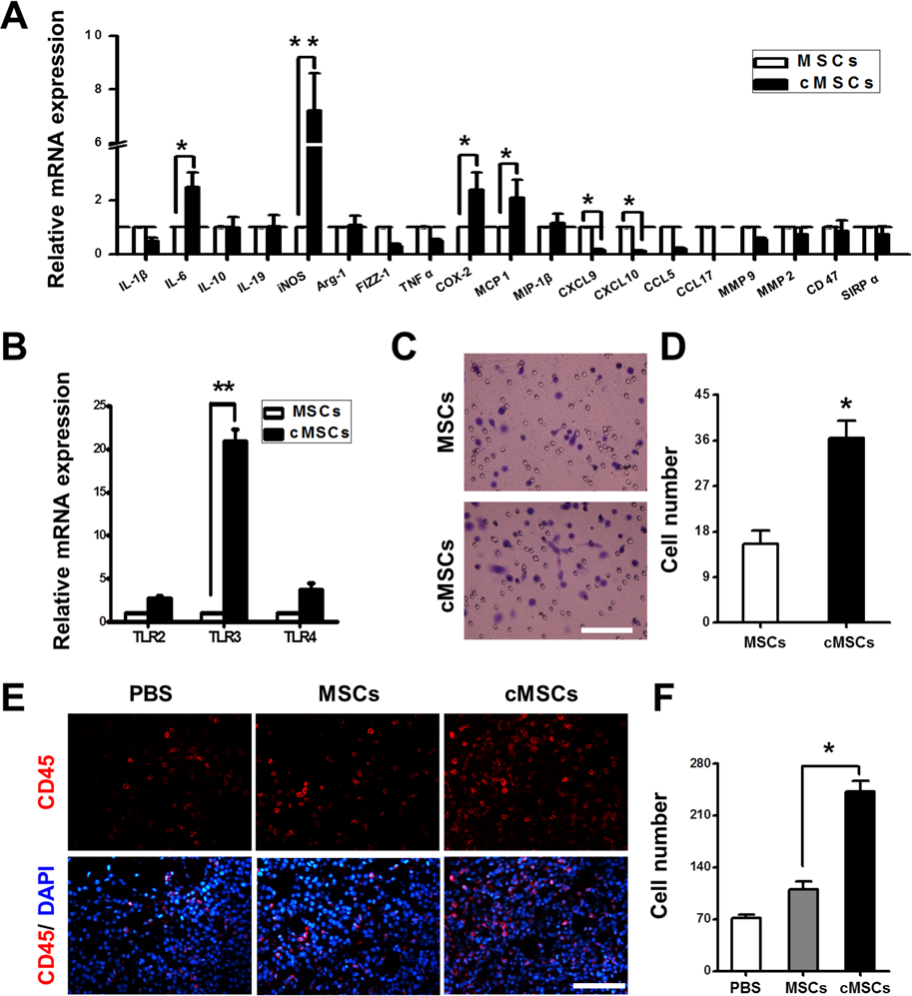
Characterization of the cMSCs (**A**) The effects of the M1-conditioned medium on the activation of various gene expressions in the MSCs were assessed by using real-time PCR. *P < 0.05; **P < 0.01. (**B**) The expression of TLRs of the cMSCs was assessed by real-time PCR. **P < 0.01. (**C**–**D**) The migration ability of the cMSCs was evaluated. (C) Representative images of the MSCs (upper) and cMSCs (lower) in response to FBS in a transwell assay. Scale bar = 50 μm. (D) Average number of migrated cells in a transwell migration assay. Results are mean values ± SEM of five different fields from four independent experiments. *P < 0.05 versus MSCs. (**E**–**F**) Tumor-associated leukocytes differ among the cMSCs and MSCs treated groups. (E) Immunohistochemical staining for CD45 (red) and DAPI (blue) in 4T1-FLUC breast tumors 14 days after co-injection. Scale bars = 50 μm. (F) A larger number of CD45 positive bone marrow cells were stained in the cMSCs implantation group than in the MSCs and the PBS injection groups. *P < 0.05 vs. MSCs group. Abbreviations: HPF, high-power field.

The MSCs affect cancer progression through a number of secreted factors triggering activation of various mechanisms. The genetic abnormalities in specific genes in the cMSCs may account for the tumor-promotion activity by the cMSCs. To investigate how the cMSCs achieve their tumor-promoting effect and how they differ from untreated MSCs, we examined the gene expression profiling of the cMSCs. Using real-time PCR, we found that the transcript levels of iNOS, MCP1, IL-6 and COX-2 were markedly higher in the cMSCs than in untreated MSCs. However, the levels of CXCL9 and CXCL10 were lower (Figure 2A). A previous study reported a new MSCs paradigm by specific TLR-priming: TLR4-primed MSC1 and TLR3-primed MSC2 [17]. We used real-time PCR to identify additional TLR genes that might be affected, and found that TLR2, TLR3, and TLR4 were induced at high levels in the cMSCs compared to untreated MSCs. Specifically, TLR3 expression was increased about 20-fold after M1 medium treatment (Figure 2B). Furthermore, we examined the chemotactic potential of the cMSCs *in vitro* using transwell migration assays, and found that the cMSCs elicited a more robust migration response than the MSCs (Figure 2C, 2D).

Cancers develop in a complex tissue environment which usually contains bone marrow derived cells. We employed CD45^+^ to identify tumor-associated bone marrow cells 14 days after co-injection with 4T1-FLUC cells and cMSCs or untreated MSCs, and found that there was a great expansion of the populations of CD45^+^ cells in tumors, an effect that was dramatically greater with the cMSCs than untreated MSCs (Figure 2E, 2F). These data suggested that the cMSCs were more effective than the untreated MSCs in recruiting CD45^+^ cells to tumor sites. In view of the immunosuppressive ability of the MSCs, we examined whether cMSCs have immunosuppressive function. The splenocytes were activated with ConA followed by expansion with IL-2 for 72 hours. We co-cultured the activated splenocytes with cMSCs or untreated MSCs. The results showed that although the MSCs could not inhibit the proliferation of splenocytes, the inhibitory effects of the cMSCs on splenocytes proliferation were significantly increased (Figure S2).

### cMSCs exert tumor promotion of the required iNOS

**Figure 3:**
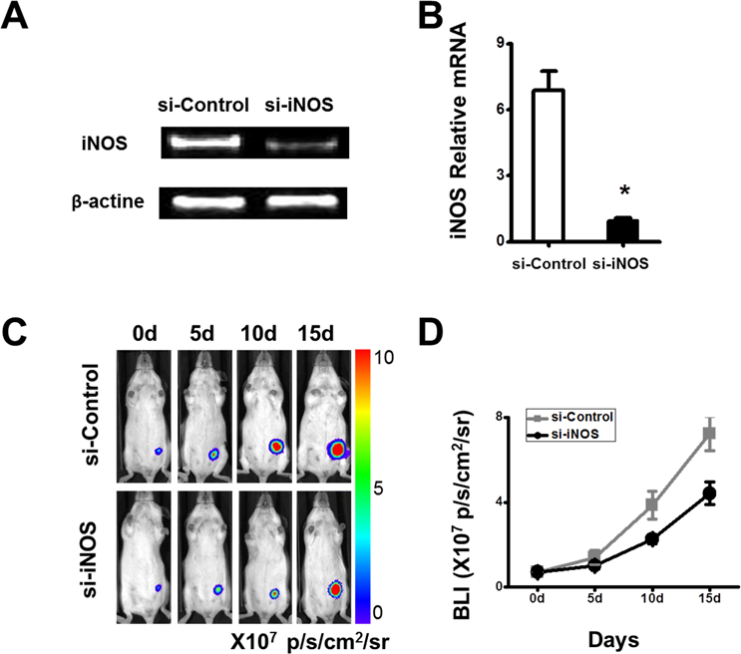
The growth of the tumor favored by the cMSCs *in vivo* can be inhibited by iNOS siRNA To determine the effect of iNOS in the cMSCs, the cMSCs were transfected with iNOS siRNA or control siRNA. Mice were divided into the si-iNOS/cMSCs group and si-control/cMSCs group. (**A**) RT-PCR showed that the expression of iNOS was decreased in the cMSCs in which transfections of iNOS siRNA were performed with a Lipofectamine. (**B**) Real-time PCR showed that the inhibitory efficiency was more than 80% compared with the control siRNA in the cMSCs. (**C**) cMSCs transfected by iNOS siRNA did not support 4T1-FLUC tumor cell growth. (**D**) Quantification of the bioluminescent signal (C) in tumors after treatments.

High concentrations of nitric oxide are known to inhibit immune responses to promote tumor growth [18]. As shown above, iNOS mRNA was up-regulated significantly in the cMSCs (Figure 2A). To investigate the role of nitric oxide, its production was shut down using siRNA to inhibit the expression of iNOS. RT-PCR and real-time PCR were performed to assess the expression of iNOS in cMSCs to further confirm the transfection efficiency of iNOS siRNA. iNOS expression was markedly decreased in the cMSCs treated by iNOS siRNA but not in the cMSCs treated by control-siRNA (Figure 3A), which showed more than an 80% inhibitory efficiency rate (Figure 3B). Moreover, in mixed co-cultures of splenocytes and cMSCs pre-treated with iNOS siRNA or iNOS inhibitor 1400 W, splenocyte proliferation was restored to a normal level (Figure S2). The results strongly suggested that nitric oxide produced by the cMSCs mediated the suppression of splenocytes. Furthermore, we demonstrated that the 4T1 tumor-promoting ability of the cMSCs was dependent upon the expression of iNOS (Figure 3C–3D). Therefore, these data supported that immunosuppression mediated by the cMSCs via nitric oxide play an important role in favoring tumor cells growth.

### MCP1-mediated macrophage trafficking is critical for the tumor-promoting effects of the cMSCs

**Figure 4:**
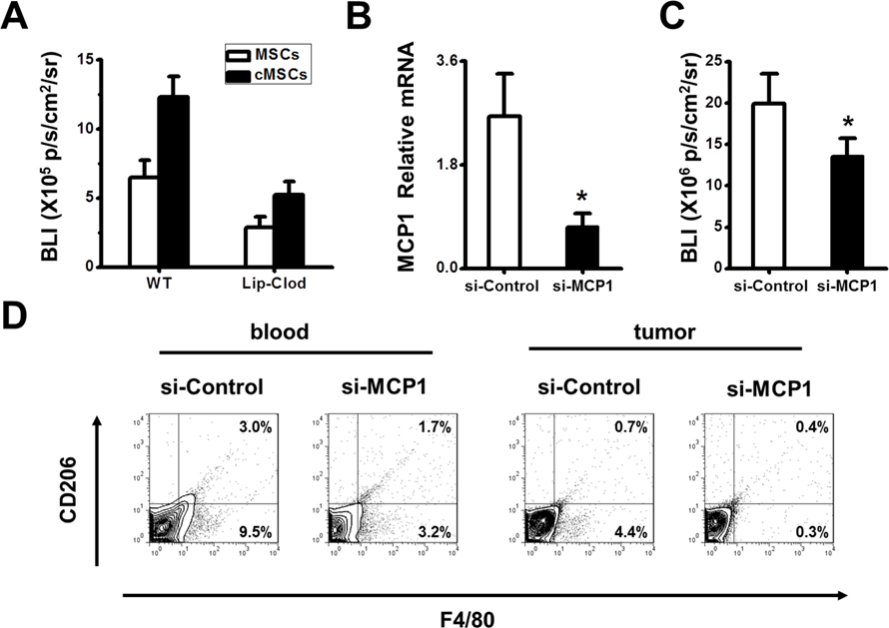
Tumor-promoting effect of the cMSCs relied on the MCP1 to recruit TAMs (**A**) Ablation of monocytes/macrophages by clodronate liposomes abolished the tumor-promoting activity of the cMSCs. (**B**–**D**) cMSCs were transfected with MCP1 siRNA. Impact of MCP1 deficiency on cMSCs tumor promoting effects was detected. Animals were divided into the si-MCP1/cMSCs group and si-control/cMSCs group. (B) Real-time PCR showed that the expression of MCP1 was decreased in the MSCs in which transfections of MCP1 siRNA were performed with a Lipofectamine. (C) The *in vivo* growth of the tumor favored by the cMSCs could be inhibited by MCP1 siRNA. 4T1-FLUC tumor cell growth was detected by BLI after establishing the breast carcinoma model. (D) Representative flow cytometry data show the frequency of CD206 macrophages from the tumor and peripheral blood of 4T1 bearing control mice.

As shown above, the cMSCs were more effective than untreated MSCs in recruiting CD45^+^ cells at tumor sites (Figure 2E), which may include monocytes/macrophages, B cells and T cells. Monocytes/macrophages are essential for the tumor-promoting effect of MSCs isolated from tumor sites [14]. To determine whether monocytes/macrophages mediated the tumor-promoting effect of the cMSCs, we depleted systemic monocytes/macrophages with clodronate liposomes (Clo-Lip) followed by injecting mixtures of cMSCs or MSCs with 4T1 tumor cells into mice 14 days after transplantation which detected the tumor growth by BLI. We found that depletion of monocytes/macrophages in our system had an effect on cMSCs-promoted tumor growth (Figure 4A), indicating that monocytes/macrophages are indispensable for tumor growth enhancement induced by the cMSCs.

MCP-1 is the CCR2 ligand. The chemokine receptor CCR2 is known to be expressed on monocytes/macrophages. Most notably, MCP1, the major chemokine for macrophage trafficking, was expressed at a high level by the cMSCs, but not by the untreated MSCs (Figure 2A), suggesting that cMSCs-produced MCP1 may play an important role in cMSCs-promoted tumor growth. To examine this possibility, we performed MCP1 knock down in the cMSCs using siRNA. We found that siRNA targeting MCP1 was capable of inhibiting MCP1 in the cMSCs (Figure 4B). To establish the role of cMSCs-expressed MCP1 in tumor growth promotion and macrophage infiltration *in vivo*, we co-transplanted MCP1 siRNA treated cMSCs and 4T1-FLUC tumor cells into C57BL/6 mice. We found that the tumor promoting activity of the cMSCs was largely MCP1 dependent since it was significantly blocked by adding MCP1 siRNA (Figure 4C). MCP1 could induce emigration of macrophages from the bone marrow to the periphery. Furthermore, the infiltrated CD206 positive macrophages in the blood and tumor sites in mice who were administered cMSCs and were treated by MCP1 siRNA or control siRNA were examined by flow cytometry. We found that an MCP1 deficiency led to a significant reduction in CD206 positive macrophages in both tumor tissues and peripheral blood (Figure 4D), indicating that MCP1 is critical for trafficking CD206^+^ macrophages. Altogether, these data demonstrate that the cMSCs probably exert their tumor-promoting effect by recruiting macrophages to tumor sites through the production of MCP1.

### cMSCs convert macrophages into M2-like cells to promote tumor growth

**Figure 5:**
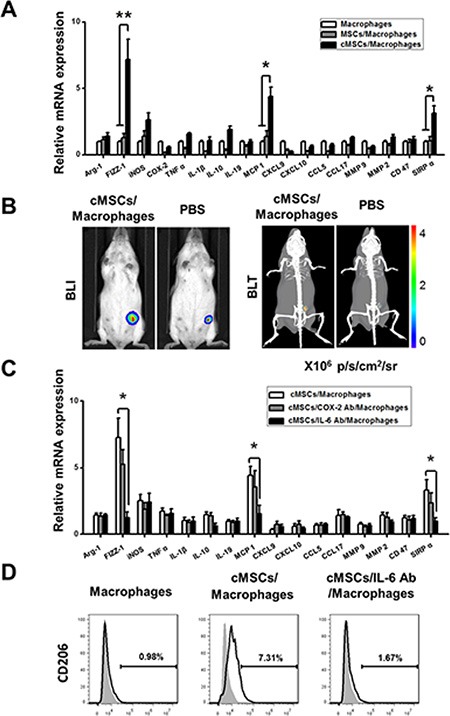
The cMSCs promoted the polarization of TAMs into M2-like cells through IL-6 (**A**) Real-time PCR showed that the cMSCs educated macrophages increased the expression of FIZZ-1, MCP1 and SIRPα compared to untreated macrophages. *P < 0.05; **P < 0.01. (**B**) The growth of the tumor was favored by macrophages stimulated by the cMSCs for *in vivo* studies. (**C**–**D**) Blocking IL-6 inhibited cMSCs-mediated induction of M2-like macrophages. (C) The effects of IL-6 and COX-2 antibody on the activation of various gene expressions in the cMSCs were assessed by using real-time PCR. *P < 0.05. (D) Characterization of the cMSCs educated macrophages treated or untreated by IL-6 antibody.

Macrophages recruited by MCP1 may polarize into an M1 or M2 subset in a tumor microenvironment. An M2 polarized population promotes tumor progression. To examine whether the cMSCs remodel the phenotype of TAM, macrophages and cMSCs were co-incubated in a transwell for 24 hours. When macrophages were further examined for their M1/M2 gene expression profile by real-time PCR, we found that macrophages co-cultured with cMSCs expressed high levels of FIZZ-1, MCP1 and SIRPα (Figure 5A), comprising an M2-preferential gene signature in comparison to control macrophages. Interestingly, these cMSCs-educated macrophages differed slightly from the traditional M2 type, since they expressed a low level of Arg-1. Meanwhile, FACS analysis showed that the increase in the frequency of CD206^+^ cells caused by the cMSCs was detected (Figure 5D). The results suggested that the cMSCs generated macrophages with a similar M2 phenotype. Moreover, to determine whether M2-like macrophages treated by cMSCs stimulate the growth of mammary cancer cells *in vivo*, we injected 4T1-FLUC cells into the mammary fat pads of mice either alone or coupled with M2-like macrophages. The accumulation of 4T1-FLUC cancer cells in the mammary fat pads was increased significantly and observed by BLI and bioluminescence tomography (BLT) at 14 days after the inoculation as compared with 4T1-FLUC cells alone (Figure 5B, Movies S1 and S2).

Previous findings showed that secretory factors play an essential role in MSCs induced polarization of M2 macrophages [11]. In this study, IL-6 and COX-2 were expressed at higher levels in the cMSCs than MSCs, thus we explored whether these secretory factors contributed to the polarization of TAM toward an M2-like phenotype. Hence, macrophages were co-cultured with cMSCs in transwells in the presence specific neutralizing antibodies for IL-6 and COX-2 for 24 hours, and M2 macrophage characteristics were determined by real-time PCR and flow cytometry. We showed that the addition of neutralizing antibodies specific for IL-6 significantly decreased the mRNA expression of FIZZ-1, MCP1, SIRPα, and reduced the percentage of CD206 positive cells, as compared with the co-culture control treated with nonspecific antibodies (Figure 5C, 5D). Therefore, these results suggested that IL-6 contributed to the induction of M2-like macrophages mediated by co-culturing with cMSCs.

## DISCUSSION

**Figure 6:**
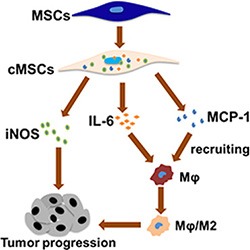
Proposed model depicted the interaction between macrophages and the MSCs in promoting tumor growth M1 macrophages could activate the MSCs to adopt a regulatory phenotype, and the cMSCs promoted tumor growth by iNOS, MCP1 and induced macrophages toward M2-like macrophages.

The inflammatory response can promote carcinogenesis; one of the major challenges in understanding the connection between inflammation and cancer is to identify the triggering events that lead to the inflammatory responses which can promote tumorigenesis. Our findings demonstrated an event of cancer development induced by inflammation: when the microenvironment became M1 pro-inflammatory, the MSCs acquired a regulatory phenotype and promoted tumor growth. The tumor-promoting abilities required iNOS and TAMs; TAMs were recruited by MCP1 and further polarized into an M2-like phenotype to affect tumorigenesis. We use Figure 6 to describe this effect. These results thus establish a mechanistic link between the M1 macrophage and tumor cells via MSCs.

The tumor microenvironment is a complex cellular, molecular network. In this network, one kind of cell may be subjected to influences from various stimuli. In our experiments, the cMSCs could become a potent immune-regulatory component. A previous study found that the concomitant presence of TNF-α and IFN-γ could induce the immunosuppressive functions of the MSCs, as these cytokine combinations provoke the expression of high levels of CXCL9, CXCL10 and iNOS [18]. In our experimental setting, CXCL9 and CXCL10 were expressed at low levels in the cMSCs. These differences may suggest that the combination of TNF-α and IFN-γ could not completely mimic the microenvironment formed by M1 macrophages.

The key factor of this tumor promoting pathway is the MSCs. Recently, the immune modulatory property of the MSCs has been extensively studied by investigators [12]. The MSCs influenced both adaptive and innate immune responses. The ability of the MSCs to control an inflammatory environment has been used in inflammatory associated disease, including myocardial infarction [19], peritonitis [20], and sepsis [21]. In addition to polarization of macrophages, the MSCs could also polarize into two distinctly acting phenotypes following specific TLR-activation including MSC1 and MSC2 [17, 22]. MSC1 was pro-inflammatory, and MSC2 showed anti-inflammatory properties. MSC1-based treatment of established tumors in an immune competent model attenuated tumor growth and metastasis in contrast to MSC- or MSC2-treated animals in which tumor growth and spread was increased [23]. In our experiments, we found that the cMSCs could significantly promote tumor growth, recruit CD45 positive bone marrow cells *in vivo*, and had immunosuppressive potential. Meanwhile, Quantitative PCR showed that the transcript levels of iNOS and TLR3 were markedly higher in the cMSCs than in the untreated MSCs. Overall, these results may suggest that the cMSCs had a regulatory-like profile similar to MSC2.

Previous reports indicated that the MSCs may be involved in cancer initiation *in vivo* and the MSCs may spontaneously transform into malignant cells *in vitro* [24]. It is clear that the MSCs secrete a number of paracrine factors that may influence tumor growth [25, 26]. However, the exact mechanisms of MSCs-mediated tumor growth are still debated, especially with regards to immunosuppression. Previous data found that the prevention of graft-versus-host disease by the MSCs was dependent on iNOS, and that bone marrow-derived MSCs could exert immunosuppressive effects by iNOS to favor B16 melanoma cell growth [18]. In our experiments, cMSCs-mediated immune regulatory effects occurred via iNOS *in vitro*. After iNOS gene knockdown, the immune suppression and tumor growth enhancement of the cMSCs were reduced. These results suggested that NO-mediated immunosuppression by the cMSCs was required in tumor progression. Additionally, macrophages are the main cells in the tumor microenvironment [27]. A previous study showed that tumor-educated mesenchymal stromal cells recruit macrophages via CCR2 [15]. In our experiments, we found that the cMSCs could recruit macrophages to tumor sites, render macrophages into M2-like cells, and consequently enhance tumor growth. Therefore, the cMSCs could create a negative-feedback loop with macrophages in the tumor microenvironment. With the presence of pathogens or tissue damage, the negative-feedback loop may be initiated by tumor cells following accelerated growth.

In the tumor microenvironment, the expression of various immune mediators and modulators as well as the abundance and activation state of different cell types dictate in which direction the balance is tipped and whether tumor-promoting inflammation or antitumor immunity will ensue. In our experiments, when exposed to sufficient levels of pro-inflammatory cytokines in the M1 medium, the MSCs responded to adopting an immune regulatory phenotype to dampen inflammation and promote tumor growth. Our research may inspire a subsequent study especially in how inflammation induces tumor development.

## MATERIALS AND METHODS

### Reagents

Recombinant mouse TNFα, interferon (IFNγ), granulocyte macrophage-colony stimulating factor (GM-CSF), M-CSF, interleukin (IL)-4, IL-6, IL-10, IL-12, IL-2 and IL-15 were from Pepro Tech (London, UK). Lipopolysaccharide (LPS) was purchased from Sigma. Monoclonal rat anti-mouse antibodies against CD29, CD44, CD45, and CD11b were from BD Biosciences; and rabbit anti-mouse inducible nitric oxide synthase (iNOS) and CD206 monoclonal antibodies were from Abcam. Alexa Fluor 594 and Alexa Fluor 488-conjugated secondary antibodies were from Invitrogen.

### Cell culture

The human breast carcinoma MDA-MB-231-FLUC cells, human HCC-LM3-FLUC hepatoma cells, human U87MG-FLUC glioma cells and murine 4T1-FLUC breast cancer cells were stably transfected with a lentiviral vector containing a firefly luciferase reporter gene were first selected *in vitro*, and then injected into immune-deficient or normal mice. Murine macrophage-like cells RAW264.7 were cultured in Dulbecco's modified Eagle’ medium (Gibco), M1-polarized macrophages were prepared by stimulating RAW264.7 macrophages with 100 IU/ml IFNγ plus 10 ng/ml LPS overnight. MSCs were collected from the bone marrow of the tibia and femurs of mice aged 6–10 weeks.

### Animal model

Female C57BL/6 nude mice (6–7 weeks old) were purchased from the Department of Experimental Animals, Peking University Health Science Center. Animal experiments were performed according to the guidelines of the Institutional Animal Care and Use Committee at Peking University (Permit Number: 2011-0039). Mice were housed in a specific pathogen-free colony in the animal facility, and 100 μl of 2 × 10^6^ MDA-MB-231-FLUC cells, or 100 μl of 2 × 10^6^ 4T1-FLUC cells suspended in saline were injected into the mammary glands of mice to establish the orthotopic breast cancer model respectively. The orthotopic liver mouse models were established by injecting 100 μl of 2 × 10^6^ HCC-LM3-FLUC cells suspended in saline into the liver of BALB/c nude mice. For orthotopic brain tumor models, 10 μl of 2 × 10^5^ U87MG-FLUC cells suspended in saline were implanted manually 2 mm anterior and 2 mm to the right of the bregma in the brains of mice. After the injection, the surface was cleaned with a sterile cotton swab, and the burr hole was filled with bone wax.

To detect the effect of the cMSCs on tumor growth, breast, liver, and brain carcinoma cells were injected either alone or coupled with the MSCs into nude mice. The MSCs were treated with M1 medium or left untreated. Tumor cell growth was evaluated by BLI. Mice were divided into three groups (*n* = 6 per group): the MSCs group, in which mice received co-injection of 1 × 10^6^ MSCs and tumor cells in PBS; the cMSCs group, in which mice received the same amount of M1 medium-treated MSCs and cancer cells; and the control (PBS) group received only PBS and cancer cells. Mice were matched for age and gender in each experiment.

To determine the effect of iNOS in the cMSCs, 4T1-FLUC tumor cell growth was detected by BLI after establishing the breast carcinoma model. Animals were divided into two groups (*n* = 6 per group): the si-iNOS/cMSCs group, in which mice received 1 × 10^6^ iNOS siRNA treated cMSCs and 2 × 10^6^ 4T1-FLUC cells in 100 μl of PBS; and the control (si-control/cMSCs) group also received 1 × 10^6^ control siRNA treated cMSCs and 2 × 10^6^ tumor cells in 100 μl of PBS. Mice were matched for age and gender in each experiment.

To examine the effect of macrophages on tumor growth *in vivo*, mice were injected with approximately 110 mg/kg of clodronate liposomes I.P. or equal volume of PBS liposomes. Then, 1 × 10^6^ MSCs or cMSCs were co-injected with 4T1-FLUC cells. Tumor cell growth was evaluated by BLI on day 14.

To determine the effect of MCP1 on cMSCs, cMSCs were transfected with MCP1 siRNA. Animals were divided into two groups (*n* = 6 per group): the si-MCP1/cMSCs group, in which mice received 1 × 10^6^ MCP1 siRNA treated cMSCs and 2 × 10^6^ 4T1-FLUC cells in 100 μl of PBS; and the control (si-control/cMSCs) group also received 1 × 10^6^ control siRNA treated cMSCs and 2 × 10^6^ tumor cells in 100 μl of PBS. Mice were matched for age and gender in each experiment.

To examine the effect of the cMSCs educated macrophages on tumor growth, cMSCs educated macrophages were co-injected with 4T1-FLUC cells, and tumor cell growth was evaluated by BLI.

### BLI

BLI was performed using the Xenogen IVIS Lumina II system (Perkin Elmer, Waltham, MA, USA) as detailed previously [28, 29].

### Real-time PCR

Total RNA was extracted from cell pellets using Trizol Reagent (Invitrogen) and treated with RNase-free DNase I (Qiagen, USA). First-strand cDNA synthesis was performed using an ABI High-Capacity cDNA RT kit (Applied Biosystems, USA). Then, RT-PCR and Real-time PCR were done. Primers used in PCR are listed in Table S1, including interleukin-6 (IL-6), monocyte chemoattractant protein (MCP1), Macrophage inflammatory protein-1β (MIP-1β), tumor necrosis factor-α (TNF-α), interleukin-19 (IL-19), matrix metalloproteinase-2 (MMP-2), arginase type 1 (Arg-1), inflammatory zone 1 (FIZZ-1), interleukin-10 (IL-10), CD47, and signal regulatory protein alpha (SIRPα). The total amount of mRNA was normalized to endogenous GAPDH mRNA. Real-time PCR was performed in triplicate with the Fast Start Universal SYBR Green Master (ROX; Roche, Mannheim, Germany) and the iCycler iQ52.0 Standard Edition Optical System (Bio-Rad, Hercules, CA, USA).

To assess the gene expression of the cMSCs, the MSCs were seeded onto 12-well plates (Corning, USA) at a density of 2 × 10^5^ cells/well and incubated overnight. The medium was replaced with a fresh M1 medium and cultured for 24 h, after which real-time PCR analysis was performed. All experiments were performed in triplicate.

To examine the gene expression of the cMSCs educated macrophages, RAW264.7 macrophages were co-cultured with cMSCs in a transwell for 24 hours. The upper part was the MSCs, and the lower portion included macrophages. Then, real-time PCR analysis was performed. All experiments were performed in triplicate.

### Immunohistochemistry

Tumors were washed thoroughly in PBS and embedded in the optimal cutting temperature medium (OCT) (Sakura Finetek, Torrance, CA, USA). Cryosections (5–6 μm) were cut and stained with antibodies according to the manufacturer's protocol. To quantify tumor-associated bone marrow cell recruitment, the tumor sections were stained with rat anti-mouse CD45 (BD Biosciences Pharmingen, San Diego, CA), and the percentage of CD45-positive cells was determined by counting the number of cells in six random fields (400× magnification) from three histology sections. Counting was performed by two “blinded” independent investigators. Alexa Fluor 594-conjugated donkey anti-rat secondary antibody (BD Biosciences Pharmingen, San Diego, CA) was applied appropriately. DAPI was used for nuclear counterstaining.

### Short interfering RNA (siRNA) synthesis and transient transfection

The siRNA sequences of iNOS (Sense: 5′-CAGCTGGGCTGTACAAACCdTdT-3; Antisense: 5′-CATTGGAAGTGAAGCGTTTCGdTdT-3′) and MCP1 (Sense: 5′-AAUUGAUUUAGCGUACACGdTdT-3; Antisense: 5′-CGUGUACGCUAAAUCAAUUdTdT-3′) were designed by using Oligoengine software and confirmed by the nucleotide Basic Local Alignment Search Tool (BLAST) searches. Transfections were performed with a Lipofectamine 2000 kit (Invitrogen) according to the manufacturer's instructions. Cells (1–3 × 10^6^) grown to a confluency of 50–60% in 10 cm Petri dishes were transfected with the siRNA sequence, and then the cells were harvested 48 hours after transfection.

### Flow cytometry

For macrophage surface marker analysis, macrophages were incubated with fluorescent labeled rat anti-mouse CD11b, iNOS, and CD206 antibodies (BD Biosciences Pharmingen, San Diego, CA).

To measure macrophage infiltration in the tumor sites and blood, the tumor cells and peripheral cells were stained with Alexa Fluor^®^ 594-conjugated rabbit anti-mouse CD206 (Abcam, Cambridge, MA) and FITC-labeled anti-mouse F4/80 (Abcam, Cambridge, MA).

### Statistical analysis

All data were expressed as the mean ± SEM. One-way analysis of variance (ANOVA) was used to determine the intergroup differences. Least significant difference (equal variances) and Dunnett's T3 (non-equal variances) post hoc tests were used for testing the differences between groups. All tests were two-tailed, and differences were considered statistically significant at *P* < 0.05.

## SUPPLEMENTARY MATERIALS FIGURES AND TABLE






