# Supplementary material for: Activation of mesenchymal stem cells by macrophages promotes tumor progression through immune suppressive effects
Source: Oncotarget. 2016 Mar 14;7(15):20934–44. doi: 10.18632/oncotarget.8064 (PMC4991502; doi:10.18632/oncotarget.8064)
Supplement: Supplementary file 1 [file oncotarget-07-20934-s001.pdf]

Activation of mesenchymal stem cells by macrophages promotes tumor progression through immune suppressive effects

Supplementary Materials

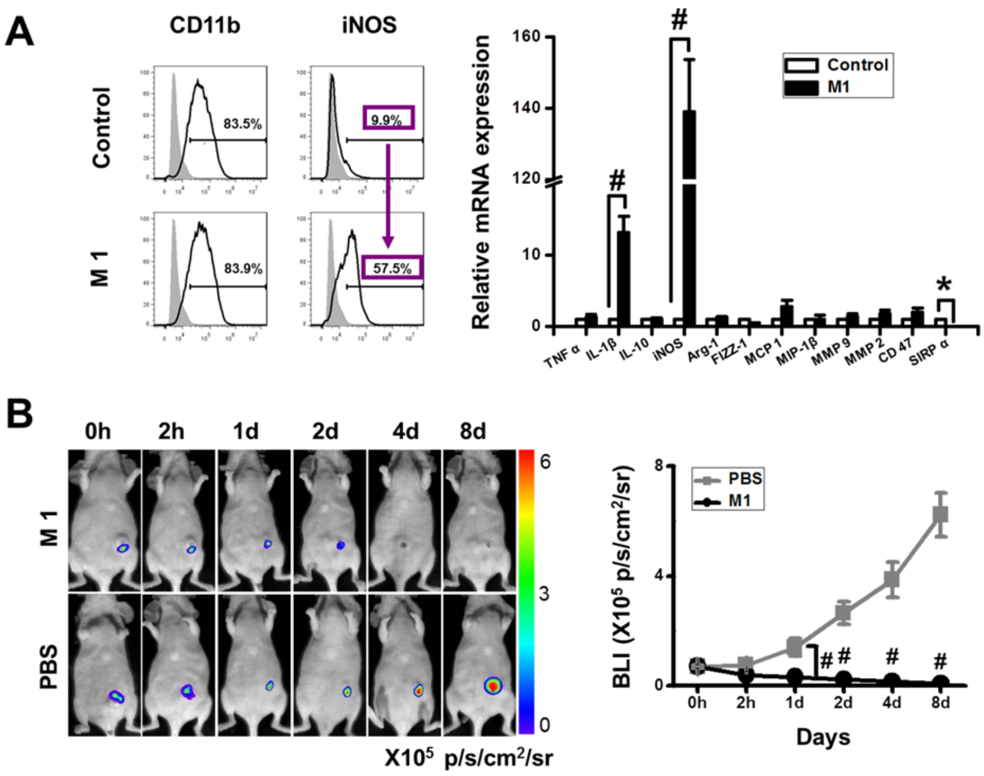

Supplementary Figure S1: The antitumor abilities of M1 macrophages. (**A**) Characterization of M1 macrophages derived from RAW264.7 by FACS and real-time PCR. *P < 0.05 vs. control; #P < 0.01 vs. control. (**B**) MDA-MB-231 D-luciferin induced bioluminescence obtained in mice treated with M1 macrophages derived from RAW264.7 or PBS. Fourteen days after the breast cancer model in nude mice was used to examine the phagocytosis of M1 macrophages by tumor cells, M1-polarized macrophages (5×10^6 cells/mouse) in 100 µl saline (M1 group, n = 6) were injected into the tumor sites. Control animals (Control group, n = 6) underwent the cancer model procedures and only saline was injected. Quantification of the bioluminescent signal in tumors after treatments showed that M1 macrophage-treated mice displayed a significant delay in the tumor growth rate. #P < 0.05 vs. PBS group.

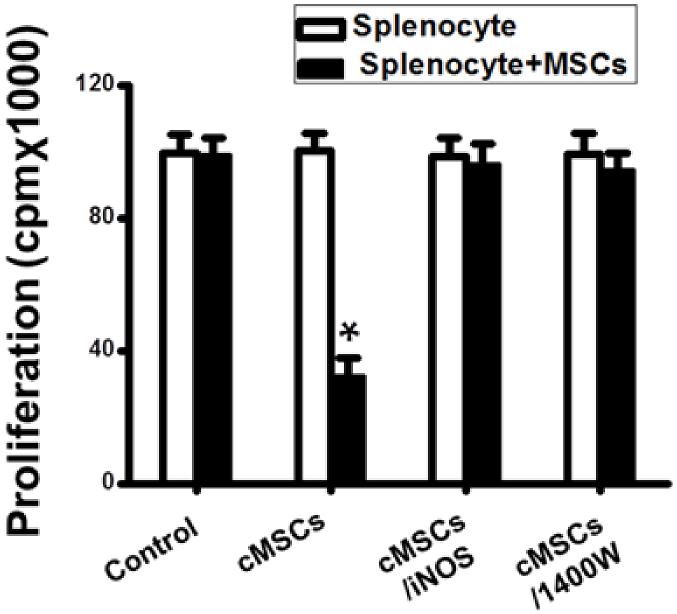

Supplementary Figure S2: cMSCs were co-cultured with fresh splenocytes plus Con A and IL-2, with or without the iNOS inhibitor-1400 W(100 μM) and siRNA of iNOS. Cell proliferation was assayed by 3H-Tdr incorporation after 72 hours.

Supplementary Movie S1: The accumulation of 4T1-FLUC cancer cells in the mammary fat pads was observed by BLT at 14 days after inoculation of cancer cells and the cMSCs stimulated macrophages.

Supplementary Movie S2: The accumulation of 4T1-FLUC cancer cells in the mammary fat pads was observed by Movie S2: The accumulation of 4T1-FLUC cancer cells in the mammary fat pads was observed by BLT at 14 days after inoculation with the cancer cells alone.

Table S1: Primers used in Real-time PCR

Primers	Forward sequence (5′–3′)	Reverse sequence (5′–3′)
IL-6	TCCCCATCTCTCATGCAGTGT	CTCTCTCCCTTCTGAGCAGCTG
MCP1	GTTGGCTCAGCCAGATGCA	CCAGCCTACTCATTGGGATCA
MIP-1β	CCCTGGGTCACTGAGTACATGA	CAAGGACGCTTCTCAGTGAGAA
TNF-α	CAGCCGATGGGTTGTACCTT	GTGTGGGTGAGGAGCACGTA
IL-19	GGTCTGGTTGGATCCCAATG	CCCATCCTTGATCAGCTTCCT
MMP-2	GGGGTCCATTTTCTTCTTCA	CCAGCAAGTAGATGCTGCCT
Arg-1	AGACAGCAGAGGAGGTGAAGAG	CGAAGCAAGCCAAGGTTAAAGC
FIZZ-1	TGCTGGGATGACTGCTACTG	AGCTGGGTTCTCCACCTCTT
IL-10	TGCTAACCGACTCCTTAATGCA	TCATGGCCTTGAGACACCTTG
CD47	CCAAACTTTCCCCAGAACAG	AGGAGGAGAAAGGAGGTTGC
SIRPα	TGCAGTTGAGAATGGTCGAA	TCCGCGTCCTGTTTCTGTA
GAPDH	ACCTGCCAAGTATGATGACATCA	CCCTCAGATGCCTGCTTCAC
